# Supplementary material for: A Stochastic Chemical Dynamic Approach to Correlate Autoimmunity and Optimal Vitamin-D Range
Source: PLoS One. 2014 Jun 27;9(6):e100635. doi: 10.1371/journal.pone.0100635 (PMC4074107; doi:10.1371/journal.pone.0100635)
Supplement: File S1 — Contains Text S1 that describes process of T-cell activation and introduction of effector and regulatory T-cells, Text S2 that describes time-dependent oscillatory behavior of antigen-specific effector (TEff) and regulatory (TReg) T cells, Figure S1 that shows impact of vitamin-D over effector and regulatory T-cell profile in presence of pathogen, Figure S2 that shows a complex representation of adaptive immune response and Figure S3 that shows a coarse-grained network of Figure S2. (DOC) [file pone.0100635.s001.doc]

**Supporting Information file for**

**A stochastic chemical dynamic approach to correlate autoimmunity and optimal vitamin-D range**

**Susmita Roy, Krishna Shrinivas and Biman Bagchi^*^**

**Text S1: Process of T-cell activation and introduction of effector and regulatory T-cells**

(1) When the pathogen enters the blood stream it is phagocytosed (that is, engulfed) by the myeloid dendritic cell in the lymph nodes, approximately about 18-24 hours after entry of pathogen. The pathogen is then cleaved with the help of lysozomes and some particular epitopes of the antigen are then transported to the surface of the molecule in MHC class II type molecule.

(2) Naive T-cells from the thymus enter the lymph nodes where they start scanning these myeloid dendritic cells. Firstly, the MHC class II type complex has a finite, short half-life and hence only a fraction of the dendritic cells present can actually function as cells capable of activating naive T cells.

(3) A T-cell which can initiate contact with the DC gets activated. This active T-cell can now activate a variety of T-cells depending on the signaling process and the co-stimulatory molecules. The probability of a successful contact is directly dependent on the [MHC−II] expressed on the surface of the dendritic cell. Now, as a DC mature, the expression of [MHC−II] increase as the pathogen is being phagocytosed. But also, since the [MHC−II] has a decay time, it can disappear from the system.

(4) It is important now to understand that DCs do not as such have a high expression of VDR and CYP27B1. When the DCs come in contact with a T-cell, the CYP27B1 expression increases and serum Vitamin D3/D_inactive_ is converted to D* . Also, the VDR expression of a dendritic cell decreases as it matures. Hence there is a competing effect on the formation of D*−VDR complex as maturation of DC on one hand leads to better contact with T cell which increases [D*], but decreases the expression of VDR. Once formed, D*-VDR destabilizes the [MHC−II] complex.

(5) Now, once the naive T-cells become activated, a series of signaling steps occurs with the help of co-stimulatory molecules and interleukins. The precise structure of these signaling, co-stimulatory molecules and interleukins decide which type of T cell is formed. Following is a small list of different type of helper T-cells and which cytokines determine formation, proliferation and inhibition. In the following we have described the important functions of different types of T-cells often categorized as effector T-cells and regulatory T-cells.

**(a) Effector T cells**: These are T-cells that promote inflammatory response by activating cytotoxic T cells or by upregulating macrophage/monocyte action.

(i) **T-helper-type1 (Th1):** These cells are commonly referred to as effector cells. They are largely responsible for resistance against intracellular pathogens. These types of cell are differentiated in the presence of Interferon-γ (INF-γ) and Interleukin 12(IL-12). The signaling process starts off through the docking of the CD40 ligands. The proliferation of fully active Th1 cells is aided by INF-[γ], IL-12 and is inhibited by IL-10 and Transforming Growth Factor Beta (TGF-β). Over-zealous presentation of these types of Th1 cells is often linked to onset of autoimmune diseases.

(ii) **T-helper-type2 (Th2):** Th2 cells mediate host defense against extracellular parasites including helminths. They are important in the induction and persistence of asthma and other allergic diseases. Th2 cells produce IL-4, IL-5, IL-9, IL-10, IL-13, IL-25, and amphiregulin. IL-4 is the positive feedback cytokine for Th2 cell differentiation and is the major mediator of IgE class switching in B cells. Both INF-γ and TGF-β are major inhibitors of Th2 type cell differentiation.

(iii) **T-helper-type17 (Th17):** Th17 cells mediate immune responses against extracellular bacteria and fungi. They are responsible for, or participate in, the induction of many organ-specific autoimmune diseases. Th17 cells produce IL-17a, IL-17f, IL-21, and IL-22. IL-21 made by Th17 cells is a stimulatory factor for Th17 differentiation and serves as the positive feedback amplifier. IL-21 also acts on CD8 T cells, B cells, natural killer (NK) cells, and dendritic cells. This type of helper cell Th17 is negatively regulated by the TGF-β .

**(b) Regulatory T cells**: These are T-cells whose main function is self-tolerance and are essential in maintaining a regulated immune response. These cells serve to downregulate the action of the effector T cells.

(i) **T_Reg_ cells**: T_Reg_ cells play a critical role in maintaining the self tolerance as well as in regulating immune responses. Increasing T_Reg_ numbers and/or enhancing their suppressive function may be beneficial for treating autoimmune diseases and for preventing allograft rejection. T_Reg_ cells exert their suppressive functions through several mechanisms, some of which require cell-cell contact. The molecular basis of suppression in some cases is through their production of cytokines, including TGF-β, IL-10, and IL-35. As mentioned above, IL-10 inhibits Th1 cell type proliferation by suppressing INF-γ production. TGF-β , on the other hand serves to suppress all forms of effector T cells. T_Reg_ cells are positively feedback regulated through the production of both IL-10 and TGF-β.

**Text S2: Time-dependent oscillatory behavior of antigen-specific effector (T_Eff_) and regulatory (T_Reg_) T cells:**

Each day each moment immune system protects us against millions of hazardous foreign elements. Whether the pathogenic stimulation strong or weak a healthy immune network always maintains a balanced regulation over the effector T-cells concentration to reduce the risk of autoimmunity. However we find that the noise aroused from T-cell dynamics could play an important role in controlling the bistablity in presence of pathogen. In particular, the cross-regulation of effector T-cells and regulatory T-cells generates a stable oscillatory dynamics that maintains body’s homeostasis. This dynamical cross-regulation has a clinical relevance in promoting the relapsing-remitting flares under autoimmune conditions. We observe that the coupled oscillation of effector T-cells (T_Eff_) and regulatory T-cell (T_Reg_) initiates within 2-5 days of time span and periodically continues (see **Figure S1**). It is important to note that both waves move in an anti-correlated fashion. The impact of vitamin-D associated intrinsic oscillatory behavior of effector T-cell often provides a dramatic signature of disease phenotype in clinical therapy [29].

**
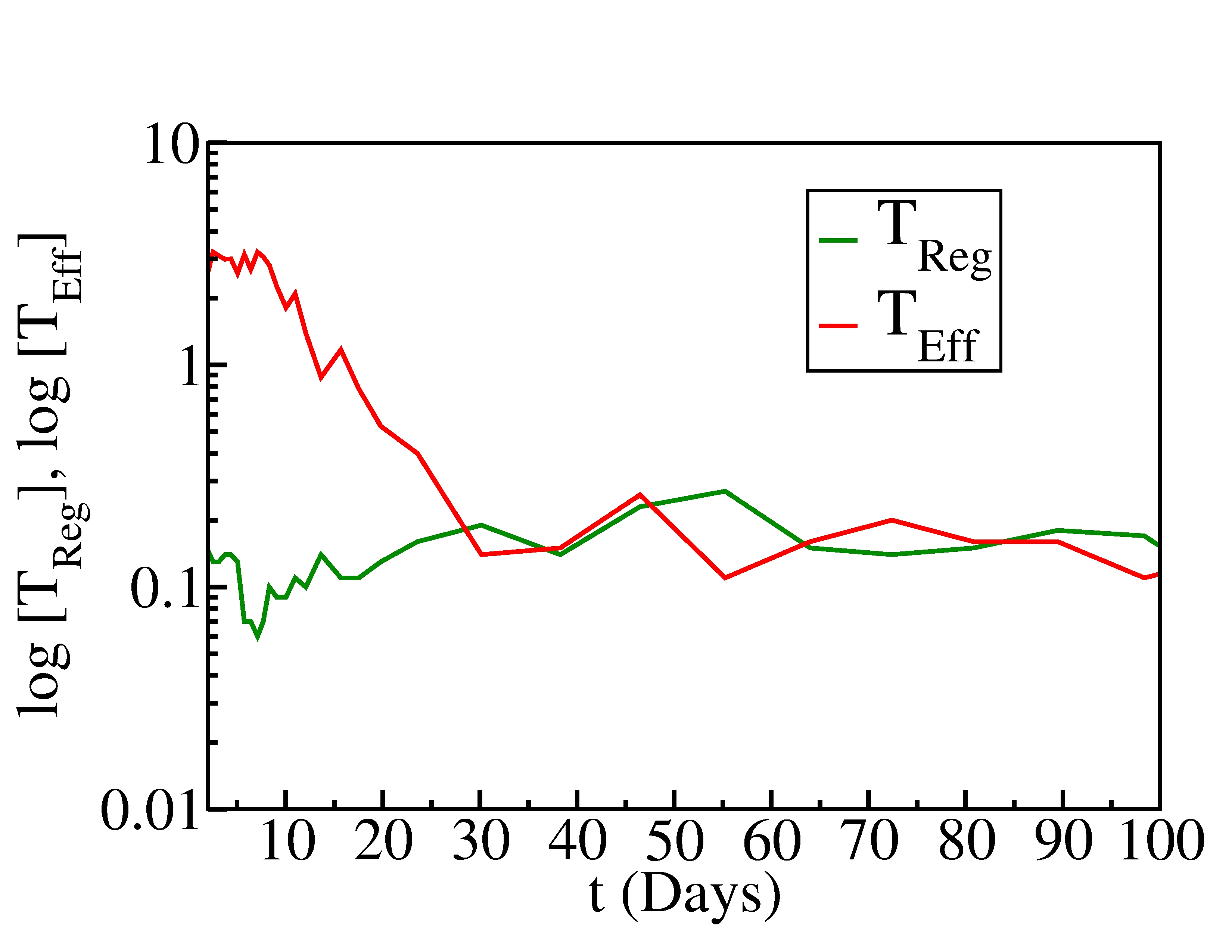
**

**Figure S1: Impact of vitamin-D over effector and regulatory T-cell profile in presence of pathogen.**

**Effector T-cells and regulatory T-cells propagate with an oscillation. This oscillation starts within 2-5days of time span and periodically continues. The coupled dynamics of effector and regulatory T-cells shows a cross-regulatory behavior. Note that the cross-regulation is important for maintaining the bistability and thus has experimental and clinical relevance. The rate parameters considered here, are similar to Figure 4 given in main text.**

**
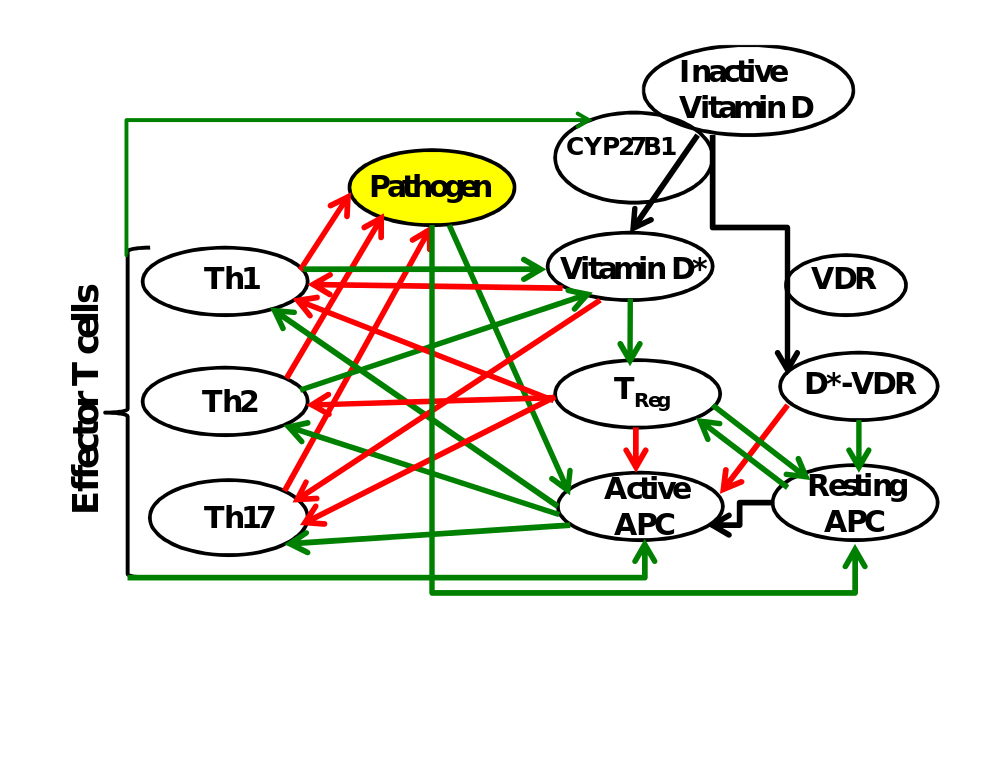
**

**Figure S2: A complex representation of adaptive immune response.**

**In this network the events are the following: (i) In presence of pathogen APC become stimulated and induce the activation of effector T-cells that ultimately destroy the pathogen. (ii) The enhanced rate of active T-cells production is regulated by both regulatory T-cells (T_Reg_) and active vitamin-D. (iii) In behind regulatory T-cell function is upregulated by active vitamin-D either through membrane initiated interaction or through VDR mediated mechanism. (iv) In addition, more regulatory T-cells lead to more resting APCs that again lead to more regulatory T-cells. Similarly more effector T-cells lead to more active APCs that again lead to more effector T-cells. We have not shown the precursor cells for APC or T-cells in this diagram. *Note that here green arrows stand for upregulation, red arrows for inhibition* and *black arrow represents conversion processes.***


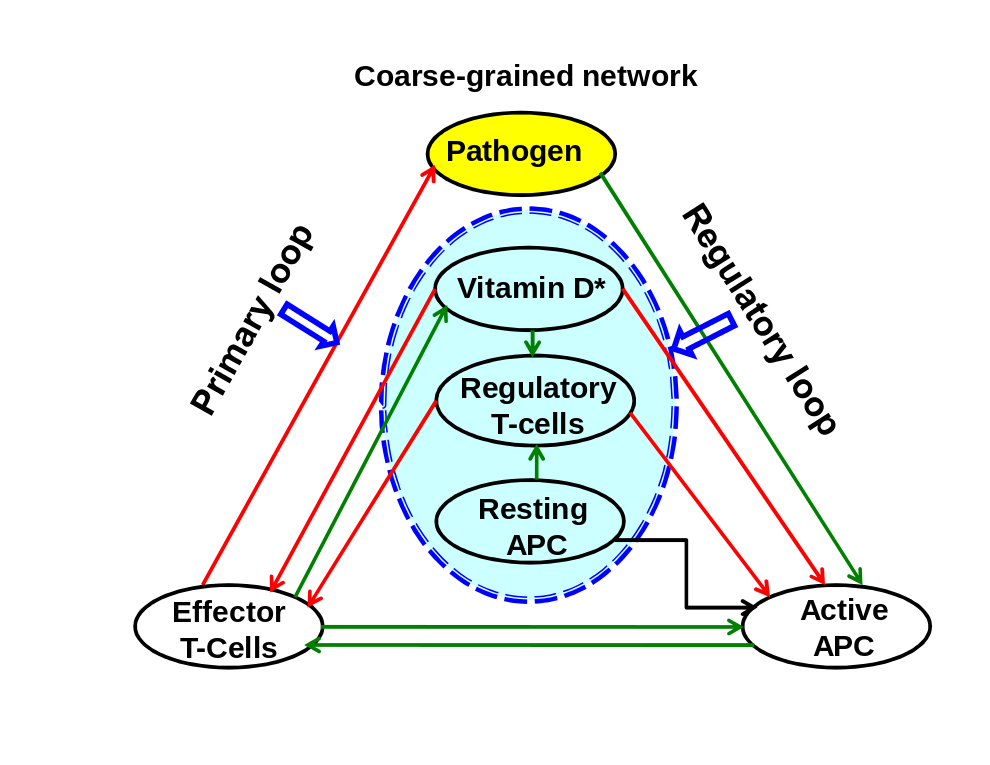


**Figure S3: Coarse-grained network of Figure S2.**

**In this diagram for simplicity we consider two loops: (i) Primary loop, (ii) Regulatory loop. Primary loop includes pathogen, active APC and effector T-cells. These components are involved in pathogen killing process. Regulatory loop have resting APC, T_Reg_ cells and vitamin-D. While regulatory loop inhibits the overexcited function of primary loop, primary loop promotes the function of regulatory loop through vitamin-D-effector T-cells interaction. Here also green arrows stand for upregulation, red arrows for inhibition and black arrow represents conversion processes.**
